# Preparation of Ce_X_Mn_1−X_O_2_ Catalysts with Strong Mn-Ce Synergistic Effect for Catalytic Oxidation of Toluene

**DOI:** 10.3390/ma18163809

**Published:** 2025-08-13

**Authors:** Zhuoxuan Zhou, Yanxuan Wang, Mingkun Cao, Zhengqi He, Rong Qiao, Fukun Bi, Yuxin Wang, Xiaodong Zhang

**Affiliations:** 1 School of Environment and Architecture, University of Shanghai for Science and Technology, Shanghai 200093, China; zzx200011060317@163.com (Z.Z.); 17205881869@163.com (Y.W.); ckwdls@126.com (M.C.); 13700149255@163.com (Z.H.); qr020607@163.com (R.Q.); bifukun@usst.edu.cn (F.B.); 2 Institute of Chemical Pharmaceutical, Taizhou Vocation & Technical College, Taizhou 318000, China; wyx790914@aliyun.com; 3 Shanghai Non-Carbon Energy Conversion and Utilization Institute, Shanghai 200240, China

**Keywords:** Ce-Mn, toluene, synergistic effect, catalytic oxidation

## Abstract

A series of Ce-doped α-MnO_2_ catalysts (Ce_X_Mn_1−X_O_2_, x = 0.04, 0.07, 0.10) were synthesized by a simple in situ hydrothermal method. It was confirmed by characterization methods such as XRD, Raman, N_2_ adsorption–desorption and SEM confirmed that the introduction of Ce significantly regulated the microstructure of α-MnO_2_, specifically manifested as the reduction in grain size, the increase in defect sites, the increase in Mn-O bond length and altered morphological structure. H_2_-TPR, O_2_-TPD and XPS analyses further revealed the strong interaction between Mn and Ce, accompanied by significant electron transfer (Ce^3+^ + Mn^4+^ → Ce^4+^ + Mn^3+^), thereby promoting the formation of Mn^3+^ species. In the test of toluene catalytic oxidation performance, C_e0.07_Mn_0.93_O_2_ exhibited the most excellent catalytic activity (T_100_ = 280 °C), while also having good thermal stability and water resistance. Furthermore, the degradation pathways of toluene were analyzed by TD-GC-MS technology: Toluene → Benzene → Benzaldehyde → Maleic anhydride → CO_2_ and H_2_O.

## 1. Introduction

Volatile organic compounds (VOCs), key precursors to secondary aerosols and photochemical smog, are significant industrial by-products posing a major threat to environmental and human health [1,2,3,4,5,6,7]. Among them, toluene, as a typical VOC, has particularly prominent hazards [8]. In recent decades, a series of technologies has been applied to the treatment of toluene, which includes adsorption [9], plasma treatment technology [10], photocatalysis [11,12,13,14], photothermal catalysis [15,16], and catalytic oxidation [17,18,19]. Catalytic oxidation has emerged as the most promising and economically efficient approach, efficiently converting toluene into low-toxicity or non-toxic small molecules [20,21]. The core of this technology lies in developing highly efficient catalysts. The main catalyst types at present are precious metals and transition metals [22]. However, precious metal catalysts face challenges in their wide application due to their high costs, susceptibility to poisoning and carbon deposits [23,24,25]. In contrast, researchers are increasingly focusing on transition metal catalysts owing to their economic advantages and superior catalytic performance [26,27,28,29,30].

Manganese oxides (MnO_x_) are considered to be excellent catalysts due to their low cost [31], readily available nature, and excellent oxidizing properties towards toluene, are considered to be excellent catalysts [32,33]. The different oxidation states of Mn (Mn^4+^, Mn^3+^, Mn^2+^) in MnO_x_ may undergo conversion between these states, which may be accompanied by the generation of oxygen vacancies [15,34]. This also implies that MnO_x_ may exhibit synergistic effects with other elements to enhance the rate of oxygen storage and release. Additionally, changes in the [MnO_6_] framework arrangement result in different MnO_2_ crystal forms, such as α- MnO_2_, β- MnO_2_, γ- MnO_2_, and δ-MnO_2_ [35]. The unique catalytic performance of these forms in toluene oxidation stems from their differing tunnel structures, surface active oxygen, and redox properties [36]. Previous studies have shown that a-MnO_2_ demonstrates relatively superior catalytic activity toward toluene oxidation. Zeng et al. [37] also proved that the crystal form of α-MnO_2_ has better catalytic performance than other crystal forms. The correlation between surface oxygen vacancies and the catalytic oxidation of toluene was also revealed through DFT calculations and characterization analyses. It was found that Mn^3+^ near the oxygen vacancies expanded the Mn-O bond through the Jahn–Teller effect [38], promoting the active oxygen in the lattice to participate in the reaction more easily. Therefore, adopting appropriate strategies to increase the content of low-valent Mn and extend the Mn-O bond is the key to further improving the catalytic performance.

As a rare earth oxide, CeO_2_ stands out as a highly promising co-catalyst owing to its superior oxygen storage capacity and reversible Ce^3+^/Ce^4+^ valence transition [39,40,41,42]. Therefore, it has been widely applied in various catalytic fields. For instance, Zhao et al. [43] prepared hollow microspheres of CeaMnO_X_ in the form of bayberries, achieving excellent catalytic performance for toluene. The introduction of Ce and the interaction between Mn were enhanced, thereby improving its low-temperature oxidation–reduction ability. Wan et al. [24] prepared the nanorod-shaped CeO_2_-MnO_X_ catalyst by the hydrothermal method and discovered that Ce^4+^/Ce^3+^ was involved in the rapid conversion between Mn^4+^/Mn^3+^, promoting the redox cycle and enhancing the ability of oxygen vacancies and oxygen migration. It can be found from this that introducing Ce into Mn can improve the low-temperature catalytic ability of MnO_2_.

At present, although Mn-Ce composite oxides have been widely applied in the field of catalysis, systematic research on regulating the crystal structure of α-MnO_2_ by in situ doping of trace amounts of Ce elements to improve its catalytic performance is still relatively lacking. Therefore, in this study, different molar Ce-doped (Ce_X_Mn_1−X_O_2_, X = 0.04, 0.07, 0.10) catalysts were synthesized using an in situ hydrothermal method, and the relationship between their doping ratio and the catalytic activity of toluene was explored, thereby screening out the catalyst with the best performance.

## 2. Materials and Methods

### 2.1. Catalyst Preparation

All the reagents used in this study were of analytical grade and no further purification was required. The catalysts of CeO_2_, α-MnO_2_, Ce_0.04_Mn_0.96_O_2_, Ce_0.07_Mn_0.93_O_2_ and Ce_0.10_Mn_0.90_O_2_ were prepared by a simple hydrothermal method. For example, take 1.18 g of KMnO_4_, 0.47 g of MnSO_4_·H_2_O and 0.33 g of Ce (NO_3_)_3_·6H_2_O. Dissolve each compound separately in 50 mL of deionized water. Stir the solutions magnetically for approximately 60 min to form uniform suspensions, and then continue stirring for an additional 30 min. The solution was promptly transferred into a 100 mL Teflon-lined stainless steel autoclave and sealed. The autoclave was placed in an oven, where it was heated at 160 °C for 12 h. After cooling naturally to room temperature, the resulting product was repeatedly rinsed with deionized water and ethanol. The cleaned product was dried at 80 °C for 12 h and then moved to a muffle furnace, with the temperature increased at a rate of 5 °C/min. They were calcined at 360 °C for 2 h, and thus Ce_0.07_Mn_0.93_O_2_ could be obtained. In addition, α-MnO_2_ was prepared by the same method and mixed with different proportions of Ce_X_Mn_1-X_O_2_ catalysts, which were named Ce_0.04_Mn_0.96_O_2_ and Ce_0.10_Mn_0.90_O_2_.

The preparation method of CeO_2_ was obtained based on [44]. Then, 19.2 g of NaOH and 1.736 g of Ce (NO_3_)_3_·6H_2_O were dissolved in 70 mL and 10 mL of deionized water, respectively. The two solutions were mixed under vigorous stirring and then vigorously stirred for 30 min. It was immediately transferred to a 100 mL Teflon-lined stainless steel autoclave and maintained at 100 °C for 24 h. After natural cooling to room temperature, it was washed several times with deionized water and ethanol, and then transferred to an oven and maintained at 80 °C for 12 h. The collected products were placed in a muffle furnace and heated at a rate of 2 °C per minute to 500 °C for 3 h, and then CeO_2_ samples were obtained.

### 2.2. Catalyst Characterization

The catalysts were characterized using a suite of analytical techniques. X-ray diffraction (XRD) patterns were obtained on a Bruker D8 Advance diffractometer (Bruker Corporation, Karlsruhe, Germany) with a Cu Kα radiation source (40 kV, 40 mA) and a monochromatic detector (Bruker Corporation, Karlsruhe, Germany), scanning at 5°/min over a 2θ range of 10° to 80°. Nitrogen adsorption–desorption experiments were performed at 77 K on a Quantachrome Autosorb-iQ-2MP system (Quantachrome Instruments, Boynton Beach, FL, USA), with samples degassed under vacuum at 105 °C for 12 h prior to analysis. Specific surface areas were calculated via the Brunauer–Emmett–Teller (BET) method, and pore size distributions were determined using Density Functional Theory (DFT) and Barrett–Joyner–Halenda (BJH) approaches. Fourier-transform infrared (FT-IR) spectra were recorded on a Thermo Fisher Nicolet iS50 spectrometer (Thermo Fisher Scientific, Waltham, MA, USA) at a resolution of 4 cm^−1^ with 64 scans. X-ray photoelectron spectroscopy (XPS) data were collected using a Thermo ESCALAB 250Xi (Thermo Fisher Scientific, Waltham, MA, USA), with energy calibration referenced to the C1s peak of carbon (284.8 eV). Raman spectra were acquired on a Horiba LabRAM HR Evolution system (HORIBA France SAS, Palais, France) using a 532 nm laser. Scanning electron microscopy (SEM) images were captured with a Thermo Fisher Apero 2C microscope (Thermo Fisher Scientific, Waltham, MA, USA) at 10 kV acceleration voltage. Hydrogen temperature-programmed reduction (H_2_-TPR) and oxygen temperature-programmed desorption (O_2_-TPD) tests were conducted on a ChemBET TPR/TPD analyzer (Quantachrome Instruments, Boynton Beach, FL, USA). For H_2_-TPR, samples were pretreated in N_2_ at 150 °C for 1 h, cooled to 50 °C, and reduced in 5.0 vol% H_2_/Ar from 50 °C to 800 °C at 10 °C/min. For O_2_-TPD, 100 mg samples were pretreated in 30.0 vol% O_2_/Ar at 300 °C for 1 h, cooled to room temperature, purged with He for 30 min, and heated from 30 °C to 800 °C at 10 °C/min under He flow. Volatile organic compound (VOC) intermediates from toluene catalysis were analyzed via thermal desorption–gas chromatography–mass spectrometry (TD-GC-MS). Gases adsorbed on Tenax-TA tubes (Perkin Elmer, Shelton, MA, USA) were desorbed at 280 °C for 10 min in a PerkinElmer TurboMatrix 350 TD (Perkin Elmer, Shelton, MA, USA), refocused in a cryo-trap, and flash-desorbed at 300 °C (40 °C/min, 6 min hold) before transfer to an Agilent 890A GC/5975C MS (Agilent Technologies, Santa Clara, CA, USA). The GC temperature program started at 40 °C (5 min hold), ramped at 5 °C/min to 180 °C (5 min hold), with MS settings of 230 °C ion source and 150 °C quadrupole.

### 2.3. Catalytic Activity Tests

The catalytic activity was evaluated in a tubular fixed-bed reactor system, which was connected to a flame ionization detector (FID) for online gas chromatography analysis. The total reactive gas mixture was prepared in the tubular fixed-bed reactor and consisted of 1000 ppm toluene, a 20.0 vol.% O_2_ balance with Ar. The gas flow rate was set at 50 mL/min (weight hourly space velocity, WHSV = 30,000 mL/(g·h)). The catalyst used had a mass of 0.1 g with a mesh size of 20–40. The waterproof performance of the catalyst was assessed under conditions where the water vapor concentration was below 20 vol.%.

Toluene conversion (X_toluene_) was calculated as follows.

X_toluene_ = (C_in_−C_out_)/C_in_ × 100% (1)

where X_toluene_ is the toluene conversion; C_in_ and C_out_ refer to the concentration of toluene in inlet and outlet gas, respectively.

The stability test was conducted under the condition of a gas flow rate of 1000 ppm toluene, 20 vol.% O_2_ balanced with Ar, corresponding to a WSHV of 30,000 mL/(g·h) at 260 °C and a reaction time of 12 h.

The water resistance test was carried out in a gas flow (1000 ppm toluene, 20 vol.% H_2_O and 20 vol.% O_2_ balance with Ar) of 50 mL·min^−1^ corresponding to WSHV of 30,000 mL/(g·h) at 260 °C and 20 vol.% H_2_O.

## 3. Results

### 3.1. Basic Physicochemical Properties of the Catalyst

**Figure 1:**
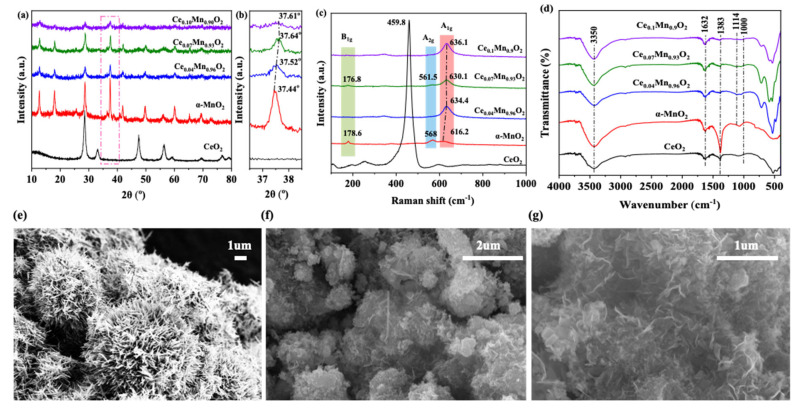
(**a**) XRD and (**b**) the partial magnification XRD patterns 2θ = 37–38°; (**c**) FT-IR and (**d**) Raman spectra of CeO_2_, α-MnO_2_ and Ce_X_Mn_1−X_O_2_ catalysts; (**e**) SEM images of α-MnO_2_ and (**f**,**g**) Ce_0.07_Mn_0.93_O_2_.

The XRD pattern of the synthesized sample exhibits diffraction peaks at 2θ = 12.8°, 18.0°, 25.6°, 28.7°, 36.5°, 37.4°, 41.9°, 49.7°, 56.1°, 60.1°, and 69.4°, as shown in Figure 1a. These correspond to the (110), (200), (220), (310), (400), (211), (301), (411), (600), (521), and (541) crystal planes of the α-MnO_2_ phase (JCPDS card No. 44-0141), respectively [45]. It is worth noting that the characteristic peaks corresponding to CeO_2_ or other cerium oxides reported by Raúl Pérez-Hernández in his previous work were not detected in the XRD spectrum [46], indicating that cerium is highly dispersed on the surface of the α-MnO_2_ matrix and does not form an independent crystalline phase. Evidence further shows that rare earth doping broadens the full width at half maximum (FWHM) while attenuating signal intensity. These changes point to a decrease in the crystallinity of the α-MnO_2_ phase, which facilitates defect formation. Evidence also suggests a relationship between crystallite size and diffraction peaks. Typically, smaller crystallites produce broader diffraction peaks with lower intensity [47]. However, compared to the other two materials, Ce_0.07_Mn_0.93_O_2_ showed higher diffraction peak intensity but narrower peak width. This may be attributed to the introduction of Ce not significantly disrupting the host structure of α-MnO_2_. The Ce_0.07_Mn_0.93_O_2_ sample exhibited the strongest diffraction intensity while maintaining the integrity of the α-MnO_2_ lattice, indicating that it possesses the highest crystallinity. Furthermore, the average crystallite size and lattice parameters of α-MnO_2_, Ce_0.04_Mn_0.96_O_2_, Ce_0.07_Mn_0.93_O_2_, and Ce_0.10_Mn_0.90_O_2_ were calculated based on the XRD patterns, as shown in Table 1. The crystallite sizes and lattice parameters of Ce_0.07_Mn_0.93_O_2_ and Ce_0.10_Mn_0.90_O_2_ were found to be smaller than those of pure α-MnO_2_, which may be due to the escape of oxygen atoms from the lattice [37]. Importantly, as shown in Figure 1b, the (211) diffraction peak of Ce_0.07_Mn_0.93_O_2_ shifts to a higher 2θ angle relative to the original α-MnO_2_, and this shift is more pronounced in other samples. According to existing literature, this shift is likely due to the replacement of larger potassium atoms (potassium atom = 1.33 Å) with smaller cerium atoms (potassium atom = 0.92 Å), leading to lattice contraction of the catalyst. This lattice contraction phenomenon is referred to as lattice distortion, and during the lattice distortion process, oxygen atoms are expelled outward, resulting in the formation of oxygen vacancies [48]. These results indicate that cerium has been successfully introduced into the α-MnO_2_ structure, and the highly dispersed cerium species have had a positive effect on the formation of oxygen vacancies [49].

The metal–oxygen vibrations in CeO_2_, α-MnO_2_, Ce_0.04_Mn_0.96_O_2_, Ce_0.07_Mn_0.93_O_2_, and Ce_0.10_Mn_0.90_O_2_ were studied using Raman spectroscopy, as shown in Figure 1c. Three characteristic peaks emerged in the spectra at 180 cm^−1^, 570 cm^−1^, and 630 cm^−1^, corresponding to out-of-plane vibrations, Mn-O chain stretching vibrations, and Mn-O vibrations perpendicular to the double-chain direction of the [MnO_6_] octahedra, respectively [8,37,50]. The absence of CeO_2_ characteristic peaks suggests highly dispersed Ce species without crystalline CeO_2_ formation, corroborating XRD results. However, as Ce doping content increased, the characteristic vibration of the Mn-O bond was affected, and the A_2g_ peak gradually shifted towards higher wavenumbers. This shift reflects [MnO_6_] octahedra formation and strong Ce-Mn interaction [51]. Notably, compared to the other catalysts, Ce_0.07_Mn_0.93_O_2_ exhibited a red shift (shift towards lower wavenumbers) in the A_2g_ characteristic peak. Generally, a higher bond order corresponds to a stronger metal–oxygen bond energy. The introduction of Ce typically weakens the Mn-O bond energy while increasing the likelihood of forming Ce-O bonds [52]. This occurs because the difference in electronegativity makes it easier for Ce to attract oxygen atoms from the Mn-O bond, leading to the formation of Ce-O bonds. This consequently weakens the strength of the Mn-O bond and introduces additional defects. Furthermore, the A_1g_ characteristic peak is significantly influenced by oxygen atoms, which may affect oxygen vacancies [53]. Therefore, compared to the other catalysts, Ce_0.07_Mn_0.93_O_2_ also exhibited a red shift in the A_2g_ characteristic peak. This shift implies that oxygen vacancies are more readily generated. The FTIR spectrum of the catalyst is shown in Figure 1d. Prominent peaks are observed at 3350 cm^−1^ and 1632 cm^−1^, corresponding to the bending vibrations of the O-H bonds in water molecules [54]. The absorption peaks below 1000 cm^−1^ are primarily attributed to metal–oxygen bonds. Specifically, the peaks near 729 cm^−1^ and 524 cm^−1^ are attributed to Mn-O bonds, while the vibration near 468 cm^−1^ corresponds to Ce-O bonds. As the Ce doping content increases, the intensity of the Ce-O peaks gradually increases, while the intensity of the Mn-O bonds gradually decreases. Among all catalysts, Ce_0.07_Mn_0.93_O_2_ maintains the optimal relative intensity of Mn-O and Ce-O bonds. To investigate the effect of Ce introduction on the surface morphology of the catalyst, we obtained SEM images of α-MnO_2_ and Ce_0.07_Mn_0.93_O_2_ samples, (Figure 1e–g). As shown in Figure 1e, α-MnO_2_ exhibits a nanorod-like structure, with its surface covered by numerous nanorod-like protrusions. In contrast, the original rod-like morphology of the Ce_0.07_Mn_0.93_O_2_ sample surface disappears, replaced by irregular plate-like structures. This morphological transformation may result from the introduction of Ce, which causes strong interactions between Ce and Mn. These interactions hinder the formation of the original nanorod-like structure, leading to the formation of irregular nanoplate-like structures. This observation is consistent with previous conclusions.

**Figure 2:**
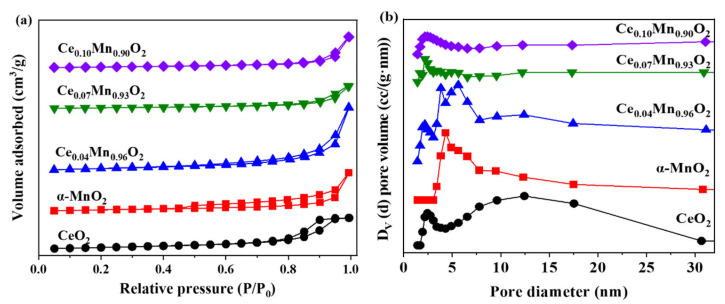
(**a**) N_2_ adsorption–desorption isotherms of CeO_2_, α-MnO_2_ and Ce_X_Mn_1−X_O_2_ catalysts; (**b**) Pore size distribution curves of CeO_2_, α-MnO_2_ and Ce_X_Mn_1-X_O_2_ catalysts.

The N_2_ adsorption–desorption isotherms and pore size results of the Ce_X_Mn_1-X_O_2_ catalyst are presented in Figure 2. All samples exhibit Type IV isotherms with H_3_ hysteresis loops, confirming the presence of mesoporous structures in these materials, as shown in Figure 2a [55]. The BJH pore size distribution curves, as shown in Figure 2b, indicate that the mesopore diameters of all materials range between 0 and 30 nm. Surface area, pore size distribution, and average pore diameter appear in Table 1**.** Notably, the mesopore diameter decreases progressively with increasing Ce doping content. This phenomenon is likely attributable to Ce incorporation into the α-MnO_2_ lattice, which may occlude surface pores of the catalyst. Furthermore, the specific surface areas of the Ce_X_Mn_1-X_O_2_ series follow this order: CeO_2_ > Ce_0.04_Mn_0.96_O_2_ > α-MnO_2_ > Ce_0.1_Mn_0.90_O_2_ > Ce_0.07_Mn_0.93_O_2_. Higher specific surface areas generally provide more active sites for catalytic reactions, thereby enhancing toluene adsorption capacity. The relatively low specific surface area of Ce_0.07_Mn_0.93_O_2_ may result from its higher crystallinity and increased structural density. While these physical properties may influence catalytic performance, they are not the determining factors for toluene catalytic oxidation. Further investigation into the catalysts’ properties is thus warranted.

### 3.2. Chemical Components and Redox Ability

**Figure 3:**
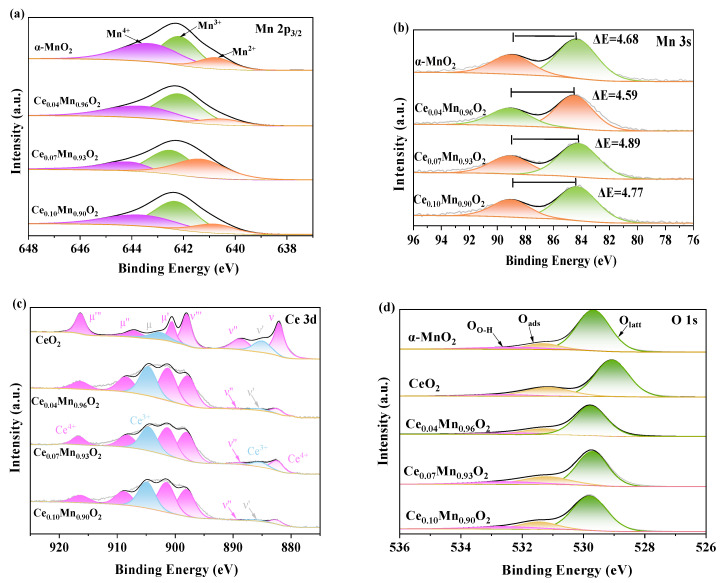
XPS spectra of CeO_2_, α-MnO_2_ and Ce_X_Mn_1−X_O_2_ catalysts: (**a**) Mn 2p, (**b**) Mn 3s, (**c**) O 1s and (**d**) Ce 3d.

The XPS is used to analyze surface information, including surface atomic concentration and the chemical state of elements, the result is shown in Figure 3. The XPS spectrum of Mn 2p_3/2_ is shown in Figure 3a, where the Mn 2p_3/2_ spectrum is divided into three parts. The peaks of Mn^2+^ are at 640.8 eV, Mn^3+^ at 642.3 eV, and Mn^4+^ at 643.8 eV [56]. The concentrations of the low-valent states of Mn (Mn^2+^ and Mn^3+^) in different catalysts are shown in Table 1. Among them, the concentration of the low-valent state of Mn in the Ce_0.07_Mn_0.93_O_2_ catalyst is the highest, reaching 80%. According to previous studies, the low-valent Mn^2+^ species and Mn^3+^ species represent indicators of surface oxygen vacancies in the catalyst. This is due to the oxygen vacancies generated during the process of Mn^4+^ changing from a high-valent state to a low-valent state to balance the charge. To further prove the existence of low-valent Mn, based on the Mn3s orbitals, the bimodal splitting (ΔEs) of Ce_X_Mn_1-X_O_2_ was calculated using the surface average oxidation state formula (AOS): AOS = 8.956 − 1.126ΔEs [57,58], where ΔEs represents the difference in binding energy between the two peaks in the Mn3s data, as shown in Figure 3b. The calculated AOS values of the prepared catalysts are shown in Table 1. It can be observed that the AOS of Ce_0.07_Mn_0.96_O_2_ shows the lowest value, which is 3.44. According to the previous results, changing the valence state of Mn and keeping it in a stable state will lead to the defect of forming asymmetric oxygen vacancies, which is consistent with the Raman results. The possible reactions that may occur are -Mn^4+^ -O_2_- -Mn^4+^ → -Mn^3+^ -O- Mn^3+^ + 1/2 O_2_ [59]. This uneven electron dispersion around the low-valent state of Mn leads to the Jahn–Teller effect [60]. This will reduce the symmetry of the [MnO_6_] octagon, resulting in structural defects. The research results show that an appropriate amount of dispersed Ce can effectively promote the generation of low-valent Mn and oxygen vacancies.

The XPS spectra of Ce 3d reveal several distinct peaks corresponding to the Ce^3+^ and Ce^4+^ species, as shown in Figure 3c. The peak position at about 883.0 (v), 889.2 (v”), 898.7 (v”’), 901.3 (u’), 907.9 (u”), and 917.3 (u”’) eV place due to the presence of the Ce^4+^ species, The peaks around 886.2 (v’) and 904 (u) eV are designated for the occurrence of Ce^3+^ species [61]. The ability to lose oxygen atoms is usually related to the ratio of Ce^3+^/(Ce^3+^+Ce^4+^), oxygen vacancies are usually related to the ratio of Ce^3+^. The proportion of Ce^3+^ in each material is shown in Table 1. Compared with Ce_0.04_Mn_0.96_O_2_ (0.260) and Ce_0.10_Mn_0.90_O_2_ (0.253), its Ce_0.07_Mn_0.93_O_2_ (0.251) shows the lowest Ce^3+^ ratio. This indicates that contact between its highly dispersed Ce atoms and Mn enhances the Ce-Mn interaction, facilitating the transfer of electrons from Ce to Mn (Ce^3+^ + Mn^4+^→Ce^4+^ + Mn^3+^) [43]. These results indicate that the strong interaction formed between Ce and Mn promotes the formation of low-valent manganese through its Jahn–Teller effect.

To further elucidate the existing oxygen species in the XPS O 1s spectrum, as shown in Figure 3d, it is evident that the O 1s spectrum comprises three distinct peaks corresponding to three different types of oxygen. Specifically, these peaks are located at 529.6 eV, 531.2 eV, and 532.4 eV, which correspond to lattice oxygen (O_latt_), adsorbed oxygen (O_ads_), and surface hydroxyl oxygen (O_O-H_), respectively [62]. The ratios of (O_O-H_ + O_ads_)/Olatt for Ce_0.04_Mn_0.96_O_2_, Ce_0.07_Mn_0.93_O_2_, and Ce_0.10_Mn_0.90_O_2_ were calculated based on the peak areas. It was observed that Ce_0.07_Mn_0.93_O_2_ exhibits a relatively high proportion of surface oxygen. Given that surface oxygen is more mobile than lattice oxygen, it replenishes the lattice oxygen consumed during the reaction more efficiently. Additionally, a significant amount of O_O-H_ was detected, which may be associated with the adsorption of H_2_O species. This suggests that the material can rapidly restore the consumed hydroxyl species during the reaction, thereby enhancing the mineralization of toluene.

**Figure 4:**
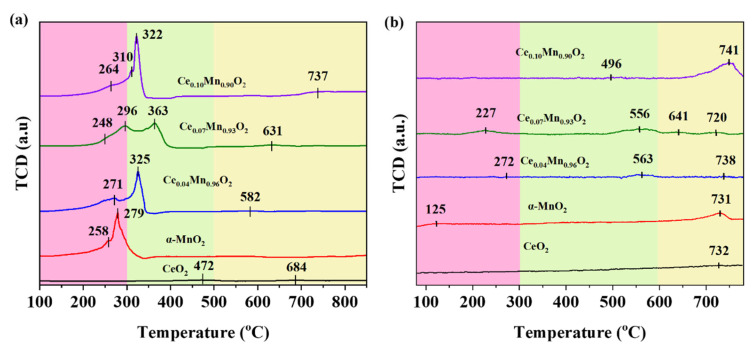
(**a**) H_2_-TPR profiles of CeO_2_, α-MnO_2_ and Ce_X_Mn_1−X_O_2_ catalysts; (**b**) O_2_-TPD profiles of CeO_2_, α-MnO_2_ and Ce_X_Mn_1−X_O_2_ catalysts.

In catalysis, the redox capacity of a catalyst is pivotal for the adsorption and activation of VOCs. The oxygen decomposition behaviors of CeO_2_, α-MnO_2_, Ce_X_Mn_1−X_O_2_ samples were analyzed by H_2_-TPR, as shown in Figure 4a. The oxygen activation capabilities of CeO_2_, α-MnO_2_, and Ce_X_Mn_1-X_O_2_ were analyzed using H_2_-TPR. It can be clearly seen that there are three reduction peaks clearly distributed in three different temperature intervals: 100–300 °C, 300–500 °C and 500–800 °C, which are marked as the low-temperature section α, the medium-temperature section β and the high-temperature section δ, respectively [58]. Among them, the peak that appears in the α low-temperature section is caused by the depletion of active oxygen adsorbed at the oxygen vacancy. It can be seen that the initial temperature of Ce_0.07_Mn_0.93_O_2_ is the lowest (248 °C). This might be due to the introduction of Ce creating oxygen vacancies, resulting in more oxygen vacancies on its surface, which is consistent with the results of XPS. The large consumption of H_2_ in the medium-temperature section is related to the transformation from Mn^4+^ to Mn^3+^, while the large consumption of H_2_ in the high-temperature section δ is related to the transformation from Mn^3+^ to Mn^2+^ [63]. which is consistent with the reduction effects reported by Raúl Pérez-Hernández in H_2_–TPR [64]. It can be found that, compared to other materials, Ce_0.07_Mn_0.93_O_2_ has lower temperature reduction peaks in the β of the medium-temperature range and δ of the high-temperature range. This indicates that it has more low-valent states of Mn, and its bulk lattice oxygen can rapidly replenish the reactive oxygen consumed by the reaction on the catalyst surface. Furthermore, according to the Wigner spin selection rule [65], it can be found that the rapid replenishment of bulk lattice oxygen to surface gaseous oxygen is the key to the catalytic oxidation of toluene.

The oxygen desorption behavior of as-fabricated catalysts was characterized by O_2_-TPD, with the results displayed in Figure 4b. It can be clearly observed that the analytical peaks have three intervals, namely the low-temperature section below 300 °C, the medium-temperature section between 300 °C and 600 °C, and the high-temperature section above 600 °C. They correspond to surface adsorbed oxygen or active surface oxygen (O_α_), surface lattice oxygen (O_β_), and bulk lattice oxygen (O_γ_), respectively [66]. It can be observed that within the low and medium temperature ranges, a distinct desorption peak can be observed at Ce_0.07_Mn_0.93_O_2_, indicating that it has a strong oxygen adsorption and activation capacity. This indicates that there are more oxygen vacancies on its surface, which is consistent with the result shown by XPS. In addition, in the high-temperature section, it can be found that it has two desorption peaks. Compared with other catalysts, its desorption peaks are more inclined to the low-temperature state. It can be seen from this that the incorporation of rare earth elements improves the oxygen storage capacity of the catalyst, and both the surface adsorbed oxygen and the surface lattice oxygen are significantly enhanced. It can be found in other materials that for Ce_0.04_Mn_0.96_O_2_, it can be observed that the temperatures of adsorbed oxygen on the surface and desorbed oxygen of surface lattice oxygen are relatively high, and no obvious desorption peak of bulk lattice oxygen is observed in the high-temperature section. However, for Ce_0.10_Mn_0.90_O_2_, although a tendency of surface lattice oxygen to shift towards the low-temperature section can be observed, However, the peak of oxygen adsorption on its surface is difficult to observe. However, a desorption peak of surface adsorbed oxygen was observed in α-MnO_2_, but the desorption peak of surface lattice oxygen was almost invisible. In CeO_2_, the presence of surface adsorbed oxygen and surface lattice oxygen was basically undetectable. Furthermore, studies have shown that lattice oxygen on the material surface is the active oxygen that first causes the oxidation of toluene. Previous studies demonstrate that while surface lattice oxygen initiates toluene oxidation, the participation of bulk lattice oxygen as well as the dynamic conversion of surface-adsorbed oxygen to surface lattice oxygen are critical for achieving complete oxidation [67].

### 3.3. Catalytic Performance of Toluene Removal

**Figure 5:**
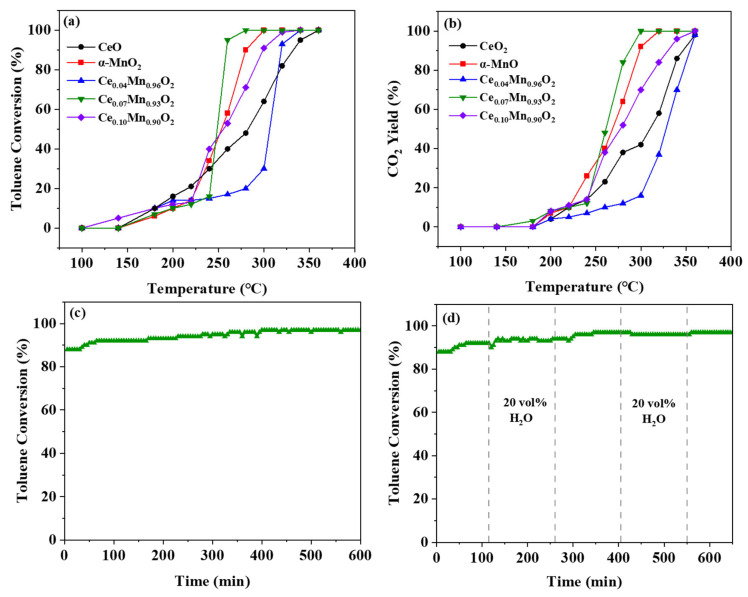
(**a**) The toluene conversions of CeO_2_, α-MnO_2_, and Ce_X_Mn_1−X_O_2_ catalysts; (**b**) CO_2_ yields of CeO_2_, α-MnO_2_, and Ce_X_Mn_1−X_O_2_ catalysts; (**c**) 12 h stability test of Ce_0.07_Mn_0.93_O_2_ catalyst; (**d**) effect of water vapor on toluene combustion over Ce_0.07_Mn_0.93_O_2_.

Catalytic toluene combustion serves as the model reaction to assess the activities of CeO_2_, α-MnO_2_, and Ce_X_Mn_1−X_O_2_ (X = 0.04, 0.07, 0.10). Figure 5a reflects the functional relationship between the conversion rate of toluene and the reaction temperature. For the α-MnO_2_ catalyst, it can be observed that the conversion rate of toluene is less than 20% below 220 °C. As the reaction temperature rises, the conversion rate of toluene increases rapidly when the reaction temperature exceeds 220 °C. The temperature at which the toluene conversion rate reaches 100% (T_100_) is 300 °C. As for CeO_2_, with the increase in reaction temperature, the conversion rate of toluene rises slowly, and the final 100% conversion rate of toluene is 360 °C. The T_100_ values on the catalysts Ce_0.04_Mn_0.96_O_2_, Ce_0.07_Mn_0.93_O_2_ and Ce_0.10_Mn_0.90_O_2_ were 360 °C, 280 °C and 320 °C, respectively. It can be found that the catalyst with Ce_0.07_Mn_0.93_O_2_ has excellent catalytic performance. This might be related to the appropriate introduction of Ce. In conclusion, it can be observed that since α-MnO_2_ itself has excellent catalytic performance for toluene, the amount of rare earth elements introduced should be moderate to improve the catalytic performance of toluene; otherwise, it will lead to a decline in performance. In addition, the conversion rate of CO_2_ at the same temperature was also studied, as shown in Figure 5b. It can be found that the conversion rate of CO_2_ is often lower than that of toluene. The reason for this phenomenon is attributed to the generation of intermediate products during the catalysis of toluene. In addition, we compared our research with the performance of other samples in degrading toluene. As shown in Table 2, it can be seen that the Ce_0.07_Mn_0.93_O_2_ sample outperforms the other samples, thus demonstrating its excellent ability to degrade toluene.

**Figure 6:**
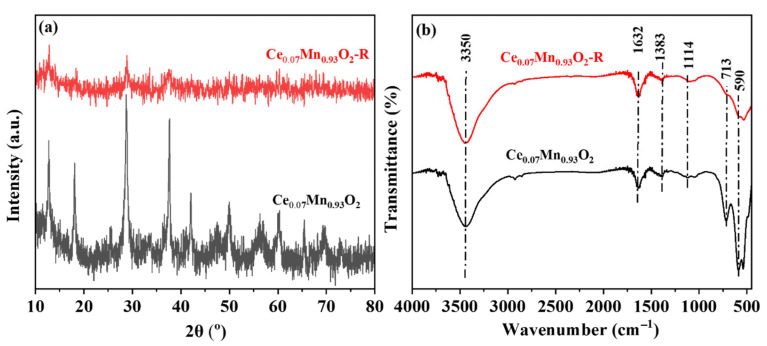
XRD patterns (**a**) and FT-IR spectra (**b**) of Ce_0.03_Mn_0.97_O_2_ before and after toluene degradation catalysts.

In addition, the thermal stability of the catalyst is an important indicator for practical industrial applications. The Ce_0.07_Mn_0.93_O_2_ catalyst was tested at 260 °C for 10 h, as shown in Figure 5c. It can be clearly observed that the conversion rate of the catalyst shows an upward trend in the initial period and remains unchanged at a conversion rate of 90%, which indicates that the catalyst has excellent thermal stability. In addition, to verify the stability of the catalyst. We conducted a series of characterization analyses on the catalyst (Ce_0.07_Mn_0.93_O_2_-R) after the reaction. Through XRD characterization analysis, it can be seen that, as shown in Figure 6a, the characteristic peaks of the catalyst after the reaction still exhibit obvious diffraction intensification and are basically consistent with those of the catalyst before the reaction (Ce_0.07_Mn_0.93_O_2_). However, through the characterization and analysis by FIRT, it can be observed that, as shown in Figure 6b, the metal–oxygen bond remains strong and shows no significant change compared with Ce_0.07_Mn_0.93_O_2_. The characterization and analysis of the two verify the integrity of its structure [68].

In addition, in order to make it more in line with the scope of practical application, we introduced 20 vol% water vapor into the reaction process during the experiment, as shown in Figure 5d. It can be found that the catalyst showed an upward trend and remained stable in the first 120 min, and then 20 vol% water vapor was introduced. Catalytic activity undergoes an initial decline upon water steam introduction, followed by a sharp rebound and subsequent stabilization. This might be caused by the fluctuation of concentration. Subsequently, another cycle was carried out, and the conversion rate of the catalyst did not show a large fluctuation range. Its conversion rate remained stable at above 90% all the time. It can be seen from this that Ce_0.07_Mn_0.93_O_2_ has excellent thermal stability and good water resistance. From this, it can be found that it has excellent practical application capabilities.

**Table 2:** Comparison of activity and water resistance between Ce_0.07_Mn_0.93_O_2_ and previously reported MnO_2_ catalysts.

Samples	S_BET_ ^a^ (m^2^/g)	Pore Volume ^b^ (cm^3^/g)	Conc.(ppm)	VOCs	GHSV (mLg^−1^h^−1^ or h^−1^)	Activity	Ref
α-MO/LMO	42.3	—	1000	Toluene	12,000	T_90_ = 260 °C	[69]
β-MO/LMO	40.0	—	1000	Toluene	12,000	T_90_ = 289 °C	
MnFe	147.92	0.540	1000	Toluene	30,000	T_90_ = 320 °C	[8]
Mn_12_Ce_1_-SW	11.2	—	1000	Toluene	15,000	T_90_ = 277 °C	[70]
0.3Cu-MnO_2_/Ni	7.88	0.041	100	Toluene	30,000	T_90_ = 240 °C	[71]
Co_1.5_Mn_1.5_O_4_	14.2	0.033	1000	Toluene	30,000	T_90_ = 267 °C	[72]
SM-4	77.3	—	1000	Toluene	36,000	T_90_ = 231 °C	[73]
Ce_0.07_Mn_0.93_O_2_	37.8	0.225	1000	Toluene	30,000	T_90_ = 258 °C	This work

^a^ BET specific surface; ^b^ Total pore volume measured at P/P_0_ = 0.99.

### 3.4. The Intermediate Products in the Toluene Catalysis Process Were Analyzed by TD-GC-MS

**Figure 7:**
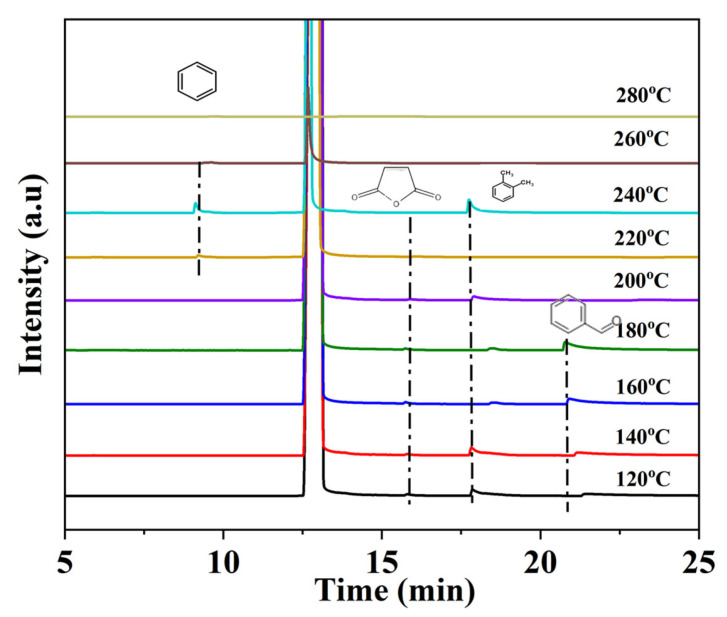
Reaction intermediates detected during the oxidation of toluene by Ce_0.07_Mn_0.93_O_2_ at different temperatures.

TD-GC-MS was employed to identify intermediates formed during toluene oxidation, as shown in Figure 7. The generation of intermediate products of Ce_0.07_Mn_0.93_O_2_ at different temperature points was displayed, and the intermediate products were marked in the figure. Analysis reveals four intermediates generated during the catalytic oxidation of toluene, namely benzene, benzaldehyde, dimethylbenzene and maleic anhydride [74]. It can be found that with the increase in temperature, the concentration of benzene shows a trend of first rising and then falling, reaching the maximum value at 240 °C. The formation of benzene may be caused by the demethylation of toluene. Meanwhile, the appearance of xylene may be due to the rearrangement or coupling effect of methyl radicals during the catalytic oxidation process. That is to say, some methyl groups are separated from toluene to generate toluene radicals, and then recombine to form aromatic hydrocarbon products containing two methyl groups. This shows that in catalytic activation, there is not just a simple demethylation phenomenon, but also the configuration adjustment of free radicals.

Additionally, it is observed that benzaldehyde appears as the temperature rises, which may be attributed to the further oxidation of the methyl group into aldehyde species. The appearance of maleic anhydride indicates that under strong oxidation conditions, not only do the side chains of toluene react, but also the aromatic rings may be attacked, thereby generating small-molecule substances. Therefore, the degradation pathways of toluene can be roughly concluded as follows: Toluene →Benzaldehyde →Benzene →Maleic anhydride →CO_2_ and H_2_O.

## 4. Conclusions

This study successfully synthesized catalysts of Ce_X_Mn_1−X_O_2_ (x = 0.04, 0.07, 0.10) with different molar ratios using a simple hydrothermal method. Among them, Ce_0.07_Mn_0.93_O_2_ maintains superior catalytic activity, thermal stability, and water resistance during toluene oxidation. When WHSV = 30,000 mL/(g·h), excellent removal efficiency for 1000 ppm toluene (T_100_ = 280 °C) is exhibited. Furthermore, Ce_0.07_Mn_0.93_O_2_ possesses an excellent crystal structure, suitable presence of low-valence Mn, and excellent redox performance. Among them, the presence of the rare earth element Ce causes a strong interaction between Ce-Mn, which facilitates the rapid conversion between Mn^4+^/Mn^3+^ and Ce^3+^/Ce^4+^. Ce_0.07_Mn_0.93_O_2_, through the rapid transformation of Ce^3+^ + Mn^4+^ → Ce^4+^ + Mn^3+^, demonstrates that it more easily forms oxygen vacancies. It exhibits higher capture of gaseous oxygen, thereby rapidly replenishing the consumed lattice oxygen. This enables toluene to be rapidly catalytically oxidized at lower temperatures.

## Figures and Tables

**Table 1:** Physical and chemical properties of α-MnO_2_, CeO_2_, and Ce_X_Mn_1−X_O_2_ catalysts.

Samples	S_BET_ ^a^ (m^2^/g)	Pore Volume ^b^ (cm^3^/g)	Pore Diameter ^c^ (nm)	Crystal Size ^d^ (310) (nm)	Lattice Parameters ^e^ (Å)	XPS Results			
						(Mn^2+^ + Mn^3+^)/Total Mn	(O_O-H_ + O_ads)_/O_latt_	Ce^3+^/(Ce^4+^+Ce^3+^)	AOS ^f^
CeO_2_	100.5	0.326	1.6–4.3 4.9–30.6	—	—	—	0.41	0.218	—
α-MnO_2_	70.2	0.379	3.0–17.5	83.6	9.86	0.56	0.24	—	3.69
Ce_0.04_Mn_0.96_O_2_	106.2	0.626	1.4–17.4	21.0	9.89	0.63	0.40	0.260	3.79
Ce_0.07_Mn_0.93_O_2_	37.8	0.225	1.4–6.5	20.3	9.79	0.80	0.51	0.251	3.44
Ce_0.10_Mn_0.90_O_2_	41.8	0.302	1.4–5.6	28.9	9.82	0.66	0.36	0.253	3.58

^a^ BET specific surface; ^b^ Total pore volume measured at P/P_0_ = 0.99; ^c^ Pore size distribution was calculated by the BJH method; ^d^ The grain size was calculated by the Scherrer Formula based on (310) crystal planes. ^e^ The lattice parameter was calculated by Jade software (jade 9) based on (110), (200), (310), (400), and (211) crystal planes. ^f^ AOS was calculated based on the XPS spectra of Mn 3s, AOS = 8.956−1.126ΔEs (eV).

## Data Availability

The original contributions presented in this study are included in the article. Further inquiries can be directed to the corresponding author.
